# Corporate social responsibility in agribusiness: climate-related empirical findings from Hungary

**DOI:** 10.1007/s10668-020-00838-3

**Published:** 2020-07-03

**Authors:** Kinga Biró, Mária Szalmáné Csete

**Affiliations:** Department of Environmental Economics, Budapest University of Technology and Economics, Budapest, Hungary

**Keywords:** Corporate social responsibility, Climate change, Adaptation, Agribusiness, Hungary

## Abstract

The corporate sector is one of the most important contributors to the global emissions of carbon dioxide and other greenhouse gases. According to a representative public opinion survey 34% of Hungarian citizens believe that corporations are responsible for climate change. The business sector is motivated to take an active role in the mitigation and adaptation of climate change. As a result, the last few decades saw a marked increase in corporate measures aiming at the reduction in greenhouse gas emissions, as well as other initiatives to tackle climate-related problems which result in numerous social issues. The aim of this paper is to assess whether Corporate Social Responsibility (CSR) can be applied as a tool in agribusiness fostering steps towards the implementation of the climate-oriented and sustainable agriculture in Hungary. The research makes an effort to explore the role and opportunities of the Hungarian agricultural sector in adapting to climate change, it also examines the extent of the mitigation and adaptation activities appearing in the CSR portfolio of the companies and what specific measures are taken to realize them. The results of the evaluation show that climate-oriented CSR activities of the companies are relatively undeveloped, but businesses are working to reduce the impact of climate change on the usage of exercising adaptation strategies. There are several actions that can help to reduce vulnerability to the consequences of climate change in the agriculture sector. The results can support not only companies but other decision-makers decisions in climate-oriented CSR activities in agribusiness.

## Introduction

Global climate change has already had a noticeable impact on the environment and socio-economic circumstances as well. Scientists have conviction that global temperatures will continue to rise, largely due to greenhouse gases produced by human activities. The Fifth Assessment Report of the IPCC has contributed with a scientific input into the Paris Agreement, which aims to limit the global temperature increase to 1.5 °C above pre-industrial levels (IPCC 2014). Achieving a 1.5 °C would require a 55% reduction in global greenhouse gas emissions by 2030 compared to 2017, and a 25% reduction for 2 °C as well. In 2017, humanity produced 53.5 gigatons of greenhouse gas emissions (UN Environment 2018). Sustainable development goals make a new framework to consider climate action. The 2019 UN Climate Action Summit reinforced that the world needs to work to achieve net-zero emissions by 2050. The Summit presented the need to increase the short-term commitments by 2020 and the mid-term commitments by 2030. Businesses showed that they are moving to take climate action. Result of the Summit, 10 regions, 93 businesses committed to reach carbon neutrality by 2050, 87 businesses have committed to implement the 1.5 °C target across their operations and value chains (UN 2019). As a result, a marked increase seen in corporate measures aiming at the reduction in greenhouse gas emissions and 19 food and agribusiness companies on eliminating deforestation, preserving biodiversity, restoring natural ecosystems and regenerative agriculture. Changes in global, regional and local climatic conditions underpinned by exposure indicators, through the changes in temperature and precipitation patterns, have a significant impact on agricultural outputs and adaptation strategies. Adaptation trends seem to be evolving, farmers are taking steps to prevent the negative effects through more precise sowing period, precision farming and more efficient species although farms have different inputs, climatic factors and soil characteristics; therefore, different adaptation procedures may be applied by different regions (Olesen et al. 2010). According to the latest IPCC Report activities during 2007–2016 globally accounted for 13% of carbon dioxide, 44% of methane and 82% of nitrous oxide emissions from human activities which is 23% of the total net anthropogenic emissions of GHG (IPCC 2018). The work of the IPCC’s Fifth Assessment Report on impacts, adaptation and vulnerability clearly shows that without proper adaptation, the effects of climate change will have a negative impact on agriculture (IPCC 2014). The need for adaptation depends primarily on the extent of climate change, geographical location and available economic, environmental and social resources. Therefore, adaptation research is one of the most dynamically developing research directions within the discipline of climate change (Bosello 2014; Porter et al. 2014; Isoard 2011; Moser 2011; Briesbroek et al. 2013). Assessing good agricultural practices and the factors that influence farmer decisions are important tasks. Adapting to the effects of climate change, environmental research and good practices have been the subject of several studies. According to scientific results, the effects to agriculture may take a very heterogeneous directions in crop production (Olesen and Bindi 2002; Chavas et al. 2009; Hatfield and Prueger 2015; Vanschoenwinkel et al. 2016) and in animal husbandry (Key and Sneeringer 2014; Qi et al. 2015). Most researches have been conducted in the Western Europe and developing countries so there is a limited amount of research available in Central and Eastern Europe focusing on the regional climate-related impacts and the special local characteristics of agriculture.

The research is also in line with the objectives of the European Green Deal (European Commission 2019a) and the new Circular Economy Action Plan as part of the Green Deal. The EU recognizes the risk of water stress, and therefore the plan encourages circular approaches to water reuse in agriculture (European Commission 2020).

In Hungary the National Framework Strategy on Sustainable Development was adopted by the Parliament in 2013 with a term ending in 2024. The strategy identifies 4 basic resources (human, social, natural and economic) that need to be preserved, enhanced and developed. The National Council for Sustainable Development (NCSD) adopted an action plan proposal on the protection of our natural heritage and the sustainable use of our natural resources in December 2019 (NCSD 2019). The proposal included the following key components for agriculture: the increase in ecological services in agriculture, the radical improvement of natural resource productivity in particular, strong development of the environmental performance of agriculture, low-carbon economy such as decrease of the emission of greenhouse gases, improvement of energy efficiency, preparation for and adaptation to the effects of climate change, mitigation of our related vulnerability (NCSD 2019). In the latest edition of the NCSD’s guide (NCSD 2020), 6 years of experience from perception and analysis have been gathered to demonstrate the objectives and targets for 2024. Significant improvements have been made in the four dimensions of sustainability since 2013, mainly in some areas of human resources (demography, poverty) and economic resources (NCSD 2020).

Hungary is located in the centre of the Carpathian Basin and is going to face with several challenges related to climate change (Bartholy et al. 2009; Torma et al. 2011; Kis et al. 2017). It is important to be prepared for the possible impacts thus adaptation can play a crucial role in sectoral and regional sustainability (Szlávik-Csete 2012; Csete et al. 2013; Szendrő et al. 2014; Bobvos et al. 2015; Csete-Szécsi 2015; Kovács et al. 2017) in Hungary. Based on the review of the international literature, numerous studies regarding climate adaptation in a Hungarian context can be found concerning agriculture (Li et al. 2017, 2018; Jolánkai and Birkás 2007; Zemankovics 2012; Gaál et al. 2014; Khanal et. al. 2019). In the case of agribusiness, the international literature often focuses more on the expected effects of climate change or agricultural GHG emissions and their reduction (Johnson et al. 2007; Smith et al. 2007; Darwin 2004; Shurpali et al. 2019) and Climate Smart Agricultures (CSAs) (Branca et al. 2011; Chandra et al. 2017; Lipper et al. 2018; Rosenstock et al. 2019; Frühauf et al. 2020; Khatri-Chhetri 2019). In agriculture reviews on sustainability assessment tools are exist (Binder and Feola 2013; De Olde et al. 2016; Marchand et al. 2014; Schader et al. 2014), whereas these assessment tools have not consistently examined which social aspects are addressed within the tools, or how these are operationalized. In fact, the evaluation of CSR activities serves a similar purpose, only from the perspective of corporate sustainability.

Hungarian projects such as VAHAVA (VÁltozás-HAtás-VÁlaszadás), Agrárklíma (Agrarian Climate), Klímahatás (Climate Impact), NATéR/AGRATéR have been dealing with the effects of climate change on Hungarian agriculture. The main objectives of these national programs were to analyse the impacts of climate change, to understand global climate change and there expected impacts on meteorological, social and agricultural terms and to formulate proposals for a national adaptation strategy (Biró et al. 2018). The main objectives of these national programs were to analyse the effects of climate change, to understand global climate change and its expected effects in meteorological, social and agricultural terms, based on which proposals and tasks were formulated for the development of a common national adaptation strategy. The Hungarian VAHAVA (“Changes-Impacts-Responses”) project, that was supported by the Hungarian Academy of Sciences and the Ministry for Environment and Water between 2003 and 2006, resulted in a breakthrough in research related to climate change in Hungary (Láng et al. 2007). The program made five recommendations to the domestic adaptation strategies (Harnos 2007) and two objectives were formulated. On the one hand to get people and economy prepare for the increased extreme weather, to bear warmer-drier periods and their impacts. On the other hand, to create and develop the organizational, infrastructural and financial conditions that will be needed for a rapid response to the harmful impacts of unexpectedly extreme weather (Faragó et al. 2010). The Agrarian Climate project is a complex study to predict the impact of climate change on agricultural production in the period of 2014–2018. The main objective of the project is to build a decision support expert system (Czimber et al. 2015). The project call attention to the implementation of sustainable agro-ecological farming along the formulated adaptation processes and measures (Neményi 2015). The Climate Impact (Complex assessment of climate change impacts) project analyse the effects of climate change on natural and agricultural ecosystems, such as mapping of soil vulnerability (Mátyás 2015), analysis of risk management options for crop production efficiency (Neményi 2015), examination of the results of sensitivity studies in maize production (Nyéki et al. 2015). The AGRATÉR project aims to extend the National Adaptation Geographic Information System (GIS) to the agricultural sector. Within the framework of the project, the most important field crops and grasslands close to nature in Hungary were examined. The results of the research point out that lower summer precipitation will cause serious droughts. These projects also show that agriculture is one of the most exposed sectors to climate change (Dockerty et al. 2005). Agriculture plays an important role in emitting GHGs and while contributing to greenhouse gas mitigation and sequestration. (Palatnik and Roson 2012).

There is far less literature on the evaluation of climate-related activities which help the large enterprises in the agricultural sector to move towards sustainability. For that reason, our survey is focusing on the assessment of the CSR activities of Hungarian agribusiness related to climate change. In Hungary, the agricultural sector plays a pivotal role in the transition towards sustainability. Moreover, nowadays it is having a priority in the COVID-19 period and its importance is unquestionable. The purpose of this paper is to give the reader an overview of mitigation and adaptation activities appearing in the CSR portfolio of domestic companies and to explain the role and opportunities of Hungarian agribusiness in adapting to climate change.

## Corporate social responsibility (CSR) for sustainable agribusiness

Several studies have examined the attitudes of large companies towards corporate integration of sustainable development (WWF 2010; Ganzi 2006; PwC 2018; Angyal 2008; AmCham 2006; Erdélyi et al. 2009). The World Wide Fund for Nature (WWF) surveyed the CSR position of the 100 largest domestic companies. Thirty-one interviews were made that revealed that companies treat CSR as an investment to create value for both parties. The authors classified the opportunities for implementing CSR into three groups: financial, in-kind and HR (WWF 2010). European Commission puts forward a new definition of CSR as “the responsibility of enterprises for their impacts on society” (European Commission, 2011). PwC (2018) made research included 729 companies. They analysed the companies reports and found that: 72% of companies mentioned the UN Sustainable Development Goals (SDGs) specified in the Agenda 2030 (UN General Assembly 2015) in their corporate and sustainability reporting, half of the companies have identified priority SDGs and 27% of the total companies mentioned SDGs as part of their business strategy. The World Bank examined how leaders and managers think about corporate social responsibility in Hungary, Poland and Slovakia. Leaders had similar views on CSR in all countries. According to the survey, the limits of CSR are the cost of implementing CSR and the lack of proper regulation (Mazurkiewicz et al. 2005).

The theory of sustainable agriculture goes back ten thousand years to early agrarian societies (Ganzi 2006). The idea of integrating CSR and sustainable agriculture only started to surface as a topic in the twenty-first century and according to Ganzi, the most agribusiness still leaves the CSR out of consideration.

Nowadays, CSR has also gained importance in agribusiness. Heyder and Theuvsen (2009) developed the “house of CSR” model which is to balance the economic, ecological and social performance of a company. Luhmann and Theuvsen (2017) focuses on consumers’ perceptions of CSR. They explore the CSR policy in German agribusiness based on Carroll’s pyramid model (Carroll 1991). Instead of model’s four classes of corporate responsibility, Luhmann and Theuvsen (2017) were identified only three CSR factors: economic, internal and external. In summary, the model cannot be confirmed for agribusiness from a consumer view in Germany.

Develop of agriculture has contributed not only to a rapid increase in food production but also to environmental and social issues (De Olde and Valentinov 2019) like GHG emissions. De Olde and Valentinov (2019) observed that the CSR initiatives which try to reconnect agriculture and society, usually cause confrontations. Agribusiness leaders understand and practice CSR, which is inconsistent with farmworkers’ living conditions and health in Mexico (Ortega et al. 2016).

Research conducted by the Corvinus University of Budapest reveals that large companies and Hungarian subsidiaries of multinational companies use CSR tools consciously (Angyal 2008). Braun and Partners revealed the CSR practice and communication of the 50 largest corporations based on Figyelő^1^ Top 200 rankings through questionnaire survey and statistical analysis (AmCham 2006). Sixty per cent of respondents (27 companies) said that CSR leads to better financial results. Erdélyi et al. (2009), 150 companies were selected from the 2007 Figyelő Top 200 rankings and their CSR communications were analysed. As a result, the Top 50 has done better in CSR communications, meaning the larger company is more aware of communicating with CSR. Corporate social responsibility can encourage and support proactive corporate behaviour that can contribute to the success and competitiveness company. With the CSR companies try to find their place in the management of present complex problems related to sustainability (Csigéné 2020).

## Agriculture’s contribution to climate change

**Fig. 1:**
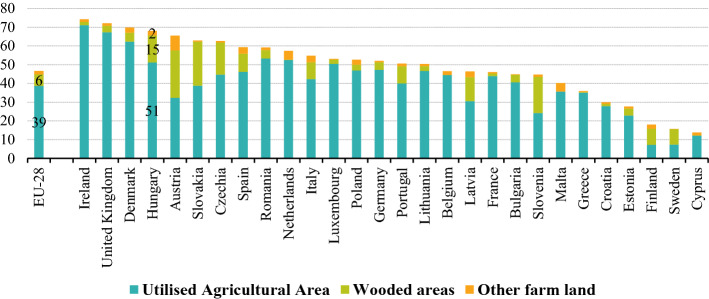
Land belonging to farms by type of land, 2016 (% share on total land area) Data.*Source*: Eurostat (2016)

Hungary's agricultural capabilities are above average based on an international comparison. Hungary produced 2% of the agricultural goods output of the European Union in 2018 (European Commission 2019b). The proportion of agricultural land (including arable land) is higher than the EU-28 average. Farms in the EU managed 39% of the total land area of the EU as utilized agricultural land, as well as wooded areas (6.2%) and other farmland not used for agriculture (2.1%) (Fig. 1).

Yohannes (2016) analysed documents on the relationship between climate change and agriculture. The documents were concluded that climate change has a strong relationship with agriculture in developing countries, because their livelihood depends on agricultural activities which mainly depend on climate. The impact of climate change is significant in developing countries because of their restricted adaptive capacity and lack of technology. Furthermore, those countries belong to the main emitters. Sustainable agriculture can help to reduce GHG emission through energy conservation, lower levels of carbon-based inputs, lower use of synthetic fertilizer. With the appropriate farming practice agriculture could be the solution for climate change by mitigation and adaptation strategies (Yohannes 2016). Land and forests have significant potential for carbon capture and storage. Non-carbon GHG emissions will always be associated with agricultural production but can be reduced through efficient and sustainable production methods (European Commission 2018). Agriculture contributes 30–40% of anthropogenic greenhouse gas emissions (Thornton and Lipper 2013). Three-quarters of agricultural greenhouse gas emissions appear in developing countries and this share may rise above 80% by 2050 (Smith et al. 2007). Although agriculture still plays an important role in the national economy, its importance continuously decreases due to the plentiful problems it faces. The increasing agricultural production has continuously increased the emissions of long-lived greenhouse gases. An Italian study provides an overview of the principal models that can be used to estimate the effects of climate change on agriculture. The classification scheme presents that a model is able to simultaneously consider many aspects related to climate change and classifying these in different classes (Salvo 2013). Aryal et al. (2019) presents a review of the impacts of climate change in the agricultural sector and adaptation options in smallholder production systems in South Asia. Agricultural production of the USA is heavily dependent on groundwater (Smidt et al. 2016). Lauer and Sanderson (2020) used a path analysis model to estimate the impact of groundwater extracted for agricultural use. Khanal et al. (2019) analysed 720 farming households in Nepal. It was found that climate change has a disadvantageous effect on agriculture, and farmers have adopted various adaptation practices to minimize the climate impacts.

The agriculture sector is the most exposed to weather extremes caused by climate change. Hungary's climate and soil conditions are excellent for high-quality, efficient agricultural production. Hungary's climate is particularly variable, as it is located on the borders of three climate zones (Atlantic, Mediterranean and Continental). Hungarian agriculture is one of the driving forces of the national economy. In Hungary the share of agricultural area, in particular arable lands, within the total area is high even in international comparison. Land use ratios within agriculture (predominance of arable land) reduce ecosystem services, and intensive production methods weaken the land productivity (NCSD 2020). In 2019, 57.1% of the total agricultural area is under cultivation (5309.5 thousand hectares) (Table 1).

**Table 1:** Agricultural area and the use of land area by land-use categories and by legal forms in Hungary. * Source*: Hungarian Central Statistical Office (KSH) database (2019a)

Year	Total land area	Agricultural area	Arable land	Orchard	Forest	Grassland	Productive land area
2013	9 303,4	5 340,0	4 325,7	92,2	1 933,6	759,1	7 375,9
2014	9 303,4	5 346,3	4 331,3	92,6	1 938,1	760,9	7 386,4
2015	9 303,4	5 346,4	4 331,7	92,2	1 939,3	761,5	7 387,6
2016	9 303,4	5349	4 332,4	92,6	1 940,7	783,2	7 376,2
2017	9 303,4	5352.3	4 334,3	93,4	1 939,3	803,8	7 370,2
2018	9 303,4	5343.8	4 333,7	94,0	1 939,7	799,3	7 355,6
2019	9 303,4	5309.5	4 317,7	94,4	1 939,5	790,4	7 319,1

All the figures are in 1000 ha (thousand hectares)

The economic role of agriculture is illustrated by its contribution of 3.6% to GDP growth (see Table 2). It is a positive change the overall efficiency of the national economy and it contributed 0.2% to the overall 4.9% economic growth.

**Table 2:** Agriculture in the Hungarian economy (%). *Source*: Hungarian Central Statistical Office (KSH) database (2019b)

Year	Share of agriculture^a^		
	GDP in production	In the investment	In employment^b^
2000	4.9	4.7	6.6
2005	3.7	4.5	5
2010	3	4.8	4.6
2015	3.7	4.8	4.8
2016	3.9	5	5
2017	3.8	4.5	5
2018	**3.6**	4.1	4.8

^a^ Agriculture, forestry, fishing

^b^ Labour force survey data

Hungary is not a significant GHG emitter. Per capita carbon dioxide emissions in 2017 were 5.2 tonnes/capita, 1.7 tonnes below the EU average. Hungary's carbon dioxide emissions have fallen by about 40% since 1990 (NÉS-2 2018), but further reductions are needed in the light of international obligations. The most important strategy for climate change is the Second National Climate Change Strategy (NÉS-2) for the period of 2018–2030, which also provides an outlook for the period 2050. The main source of GHG emissions come from the energy,^2^ industrial,^3^ agriculture and waste sector. The sectoral distribution of the country's GHG emissions depends largely on the economic structure. In 2017 total emissions of GHGs in Hungary were 63.8 million tonnes of carbon dioxide equivalents (CO2-eq) excluding the Land Use, Land-Use Change and Forestry (LULUCF) sector. Taking into account also the mostly carbon-absorbing processes in the LULUCF sector the net emissions of Hungary were 58.3 million tonnes CO2equivalent in 2017. Being about 6 tonnes the Hungarian per capita emissions are below the European average (the EU average is 9 tonnes per capita). The most important GHG is carbon dioxide accounting for 78% of total GHG emissions. The main source of CO_2_ emissions is the burning of fossil fuels for energy purposes, including transport and households.

**Fig. 2:**
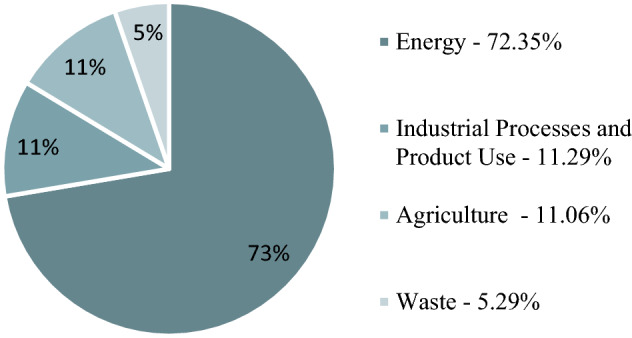
GHG emissions by sub-sectors (kt CO2 equivalent) in Hungary (2019). Data. *Source*: Hungarian Meteorological Service (2019)

The largest emitting sector was the energy sector^4^ contributing 72% to the total GHG emission in 2017. Agriculture was the third-largest greenhouse gas emitter in Hungary with 11% (Fig. 2). The three most important greenhouse gases are carbon dioxide, methane and nitrous oxide. Agricultural activities mainly produce methane and nitrous oxide emissions. Methane represents 12% in the GHG inventory while nitrous oxide contributes 7% to Hungary’s total GHG emissions. The majority of Hungary's nitrous oxide emissions (87% of total N_2_O) were generated in agriculture in 2017. Emissions from agriculture (use of fossil fuels, fertilizers, cultivation) have decreased by 41% between 1985 and 2017 (Hungarian Meteorological Service 2019).

## Agriculture’s environmental aspirations in Hungary based on a public opinion survey

**Fig. 3:**
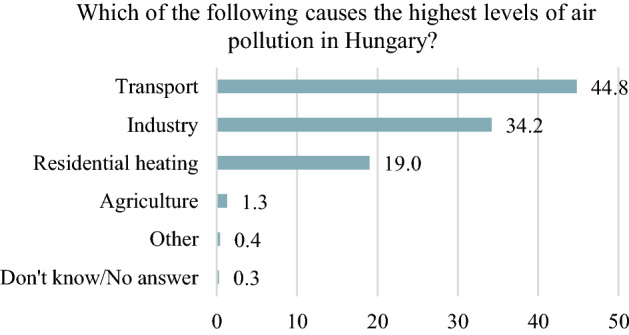
Air pollution in Hungary. *Source*: Author’s editing based on Századvég’s data (database: 1000 citizens)

The following survey represents the relevant results and conclusions of the public opinion survey carried out in June 2019 by the Századvég Foundation. The dataset used for this study comes from an opinion survey of 1000 randomly selected adult citizens in Hungary using CATI (Computer—Assisted Telephone Interviewing) methodology. The data reported in the analysis may differ by up to minus 3.1 percent points from the result of the sampling if we had asked all the adult population in the country. The survey shows the agriculture's environmental aspirations, its pollution and its contribution to climate change. In general, most people surveyed accept that climate change is a reality and is at least partly caused by humans and are concerned about it to some extent. According to the survey, almost all people consider climate change is "very important" (73%) or "more important" (25%). It also found that majority of the people believe that the transportation and industry are the most polluting sectors in Hungary (Fig. 3.). Seventy-two per cent of the emissions come from the energy sector which includes transport. In contrast, the industrial process and product use are responsible for only 10% of CO_2_ emissions.

**Fig. 4:**
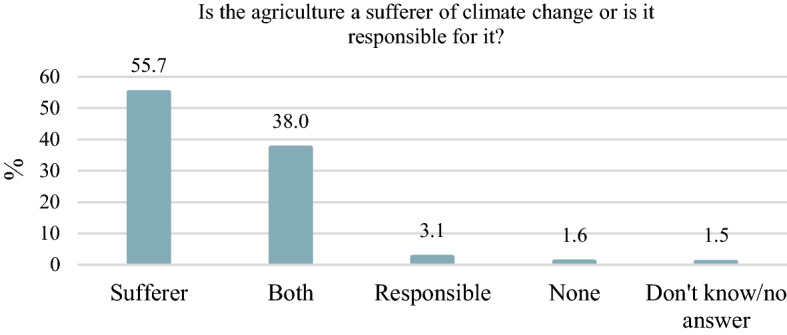
Agriculture and climate change in Hungary. *Source*: Author’s editing based on Századvég’s data (database: 1000 citizens)

The poll found that most people (56%) consider agriculture to be the sufferer of climate change and 3.1% consider it to be responsible for it while 38% can imagine agriculture in both roles (Fig. 4). Agriculture is both a victim of and a contributor to climate change.

Agricultural activities contribute significantly to emissions due to the intensive agricultural practices, the use of chemical fertilizers, pesticides and animal wastes (etc.). The emissions include nitrous oxide, methane and carbon dioxide which contribute to climate change, so they have a long-term impact on the sustainability of the agricultural sector.

## Methodology

Corporate communication representing solutions to climate change generates mostly positive emotions. Businesses could take advantage by becoming leaders in sustainability and creating a positive, climate-friendly image for their companies. For this research, a special company group was chosen. These companies have taken the lead in communication on sustainable development and climate protection. Large companies were assessed appropriately in this regard. Micro-, small and medium-sized companies interested in organic production are expected to cause less environmental impact, and they operate more sustainably than large companies, but their communication activity is negligible, because they generally do not have the financial resources in Hungary to communicate about sustainability and climate. The environmental impact of large companies is significant, so it is certainly justified to investigate in this field. The research was carried out on the top 500 databases of HVG (Heti Világgazdaság) (2017). HVG is the leading weekly economic and political journal in Hungary. The database lists the 500 largest companies in Hungary. We also compared the companies to the Deloitte Central Europe Top 500 report (Deloitte 2016) which lists 67 large corporations in Hungary. The 2016 edition of the Deloitte CE Top 500 report ranks the largest companies from the 18 countries from Central Europe and Ukraine. The ranking is compiled based on consolidated company revenues for the fiscal year ending 2016. The empirical results were obtained from two sources. On the one hand, the information was collected from a structured content analysis of the websites of the headquarters and subsidiary companies. In the content analysis, we attempt to answers the following issues:

- Do the company's website address issues relate to sustainability and climate protection?
- Does the company have a sustainability report?
- Does the report is independent or integrated?
- What topics are covered on the website?
- Do the parent company's website address issues relate to sustainability and climate protection?

On the other hand, the research based on a publicly available non-financial (sustainability) and CSR reports. The study examined the annual reports, sustainability, CSR reports, EMAS environmental statements, climate protection policies, strategies and other climate-related communication interfaces, documents and data of headquarters and subsidiary companies in 2019. While analysing the reports, the following assessment criteria were observed: the existence of CSR activities, the existence of a climate strategy, the difference between headquarter and subsidiary company climate communication, measuring and monitoring of climate objectives, top management commitment to climate objectives, the context of climate objectives: regional, national, international, global, standardized sustainability report. Based on this evaluation the climate-oriented level of CSR activities can be examined in the Hungarian agribusiness among the most significant companies also fostering moving towards sustainability.

**Fig. 5:**
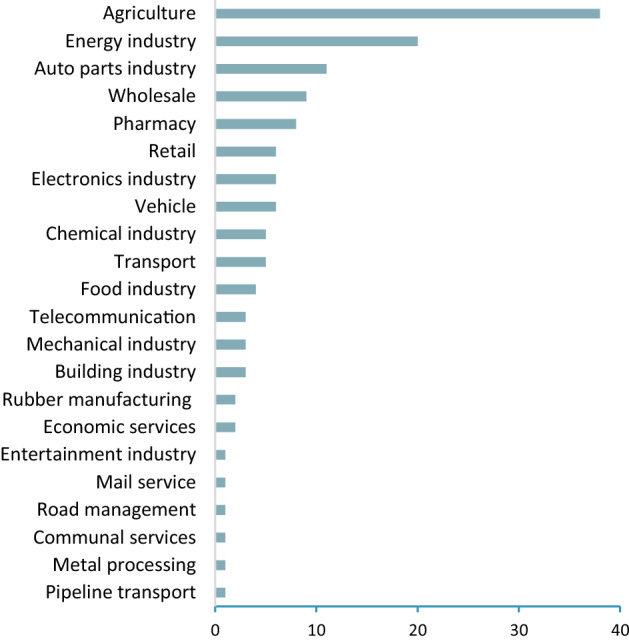
Top companies by industries. Base: all companies (*N* = 137)

## Results

The research covers the first 137 domestic companies on the top 500 list (based on net sales) published in HVG. More than half of the companies are represented by the automotive, energy and agricultural sectors (Fig. 5). Agriculture holds many opportunities to be a major force for adapting climate change. The challenge for the agriculture sector is how to reduce GHG emissions while keeping pace with the growing global demand for food and energy.

**Fig. 6:**
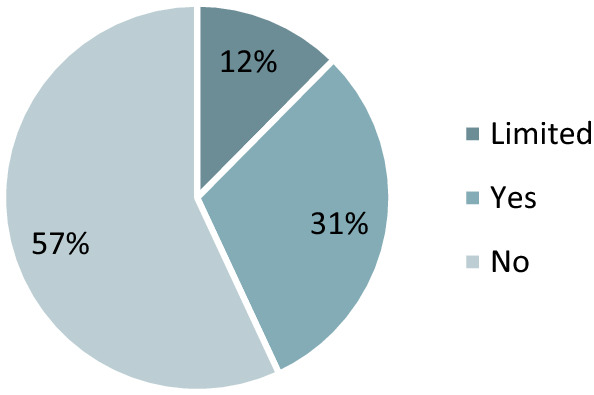
Does the company mention their CSR activity? Base: all companies (*N* = 137) Limited: Just the headquarter company

**Fig. 7:**
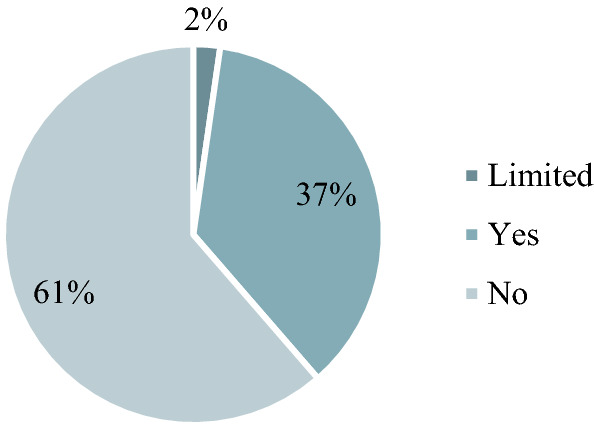
Does the company mention their CSR activity? Base: agribusiness (*N* = 44)

The environmental consciousness and CSR are becoming increasingly prominent at the company level. The analysis of the websites shows that 43% of companies (39% agribusiness) communicate their CSR activities (Figs. 6, 7). The most common tools are the websites. That followed by participation in various events, sponsorship, participation in conferences.

According to our research, corporate climate policy measures (both mitigation and adaptation) can be considered and evaluated as a sub-system of CSR. Considering the measures undertaken by companies it is useful to make a distinction between:

- CSR measures aimed at mitigation to climate change;
- CSR measures aimed at adaptation to climate change.

The most common CSR activities are as follows: circular ecosystem, precision farming, pollination program, organic farming, energy efficiency measures, sustainable supply chains, conservation of water resources, sustainable waste management, technological development (irrigation management), system development (irrigation, forecasting), consumers responsibility and needs (Table 3).

**Table 3:** The identified CSR activities in agribusiness (*N* = 44)

CSR activities	Mitigation	Adaptation
Sustainable development	Renewable energy	Investment intangible assets
Responsibility for the consumers	Reduction in pollutant emissions	Irrigation development
Energy efficiency measures	Waste discharge	Water protection investments
Technological developments	Efficient use of resources	Water damage
System development	Circular ecosystem	Plant protection
Organic farming	Conservation of protected areas	Forest protection
Circular ecosystem	Biodiversity	Organic farming
Environmentally conscious behaviour	Rehabilitation	NATURA 2000
Education, employee responsibility	Reduce environmental impact	Cooperation
Weather guarantee for products	Innovative technologies, products	Risk management
Food avalanche program	Energy efficiency programs	Food safety
Pollination program	Attitude formation	Green farming
Sustainable supply chains	Energy awareness	Sensitive areas
Investment intangible assets		Genetic research
Forestry development		Weather guarantee
Cooperation		Support for producers' investments
Preventive measures		
Environmental impact reduction		
Habitat protection		
Employee welfare		

The literature names a large number of adaptation techniques. We use the adaptation technical division developed by Dolan et al. (2001). They identified three dimensions of adaptation:

- management adaptation: based on the application of existing tools and techniques (e.g. modification the sowing time);
- technological adaptation: adaptation also requires investment (e.g. irrigation development);
- financial adaptation: management risks with financial instruments.

In the case of the examined agricultural companies, adaptation techniques were classified according to the Dolan typology (Table 4).

**Table 4:** Identified elements of managerial, technical and financial adaptation in agribusiness (*N* = 44)

Management adaptation	Technological adaptation	Financial adaptation
Green farming	Irrigation development	Risk management
Sensitive areas	Water protection investments	Food safety
Genetic research	Water damage	
Weather guarantee	Ice-frost protection development	
Support for producers' investments	Ventilation/cooling development	
Diverse cultivation system adapted to the area	Ground-water protection operations	
Reducing the use of chemicals		
Soil moisture		
Conservation of local water resources		
Protection of wildlife, biodiversity and landscape		
Cereals adaptable to climate change		
Breeding biotic and abiotic resistance		
Development of plants protection		
Water supply security in vulnerable areas		
Water protection plans		
Local water assessment		
Vulnerability assessment		

In the publishing of companies with significant national economic performance we analysed senior management commitment, policies, climate action plan, risk management and stakeholder engagement. We have addressed these issues, because we believe it is important for companies to act in the context of climate change, and to inform their stakeholders clearly and openly about their long-term strategy, goals, operations and their impacts.

Climate communication can create a positive image of the company, so it is in the company’s interest to communicate its activities related to climate change and carbon emissions and their effects on stakeholders. We examined whether companies have a specific, quantified climate protection goal. It can be judged by this how important does the company consider climate change. An ideal climate target is a long-term, time-bound targets for reducing the emissions and it is a numerical target (this can be an emission reduction targets compared to a base year or total emissions in absolute terms). Like NÉS-2 (Second National Climate Change Strategy), the short-term refers to the period 2018–2020, the medium-term to the period 2021–2030 and the long-term to the period after 2030 and the outlook to 2050. The publicly available reports show that only 44% of the 137 companies and 14% of the agribusiness have a climate protection target (Table 5). I considered the following to be relevant to the topic of climate protection: the appearance of a specific, quantified climate protection goal in the company documents. This does not mean that the other companies do not carry out such activities but do not have a long-term, well-founded and communicated commitments.

**Table 5:** Company's climate protection goals in public corporate documents

	All company	Valid percent	Agribusiness	Valid percent
Yes	19	14%	2	5%
Limited^a^	41	30%	4	9%
No	77	56%	38	86%
Total	137	100%	44	100%

^a^Just the headquarter company

**Fig. 8:**
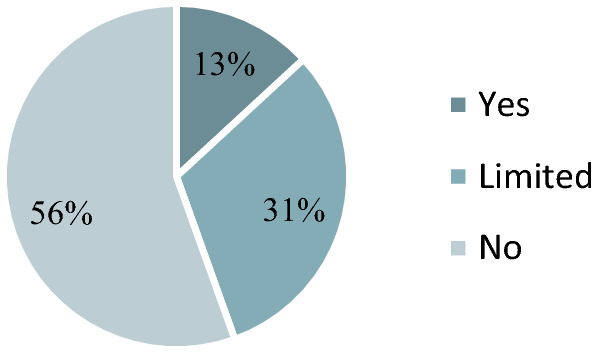
Monitoring of Carbon Targets. Base: all companies (*N* = 137) Limited: Just the headquarter company

**Fig. 9:**
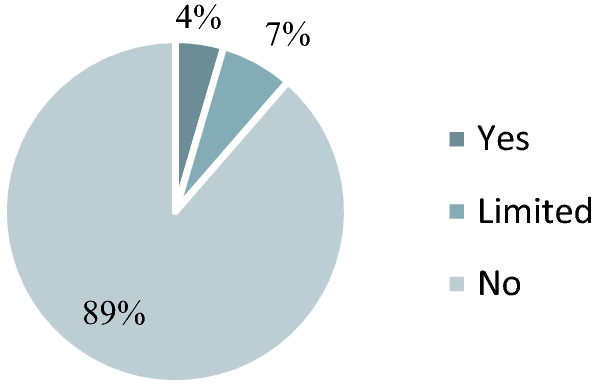
Monitoring of carbon targets. Base: agribusiness (*N* = 44)

The majority of companies (43%) set a climate protection target for carbon dioxide and 17% of domestic companies (25% of the headquarters company) also link it to a year. Companies are making increasing efforts to determine and reduce the amount of CO2 emissions from GHG. Similar to the research by Barna and Gelei (2014) we looked at how many companies measure and monitor the fulfilment of carbon reduction targets. This means that a company uses a scientifically accepted methodology to declare the exact amount of CO2 emissions associated with its activities, while taking into account the climate protection targets set by the company, measuring its emissions regularly and interval. Most of the companies (13% domestic and 31% headquarters company) are monitoring the climate targets, 11% of the agricultural companies are monitoring their climate targets (Fig. 8 and Fig. 9). The emission data are based on the methodology of the Greenhouse Gas Protocol (GHG Protocol) and uses official, internationally published emission factors.

Those companies who are monitoring the carbon emissions, 76% of the companies shown a declining trend in CO2 emissions. According to a global warming potential (GWP), the companies use carbon dioxide equivalent as an indicator. CO2 emissions data are collected in a format recognized by international standards (GHG Protocol) based on “scopes” or calculated using local methods. Only one case was in our sample where the parent company calculates CO2 data according to its methodology. Regarding to the greenhouse gas emissions, three types can be distinguished (Schaltegger and Csutora 2012) and are referred to as a GHG Protocol scope1, scope2 and scope3.

The research makes an effort to review the nature and extent of climate change vulnerability, not only in their narrow environment, but also taking into account the entire value chain, supplier side, and innovation potential for mitigating climate change that companies can incorporate as business opportunities or an operational risk. Only 14% of companies dealing with the impact of climate change on business: in the remaining 86% of companies nobody treats the risks and vulnerabilities of climate change.

**Fig. 10:**
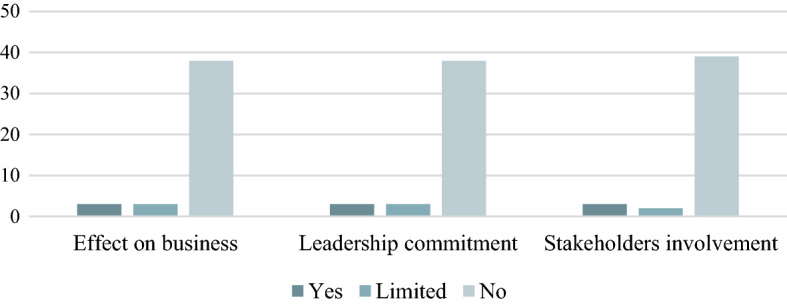
Agribusinesses Motivation (*N* = 44)

Companies have different motivations for mitigating climate change. In particular, cost reductions, regulatory compliance, new business opportunities and customer satisfaction are observed. The commitment, responsibility and example of a senior manager are essential for successful responsible corporate governance and sustainable corporate operation. Sustainability becomes a core element of a company if management is committed to it. Seven per cent of subsidiary companies and 7% of headquarters are committed to climate change at the management level (Fig. 10). A leader committed to achieving climate goals produces a sustainability report more often than a leader who does not show a commitment to climate change in public company documents, only 11% of subsidiary companies are involved stakeholders in their climate action. The results can support not only company but other decision-makers decisions to underpin the relevance of and potential in climate-oriented CSR activities in agribusiness to be able to enhance sustainability also on local, regional and global levels.

## Conclusion

Corporate Social Responsibility generally is a complex and multi-dimensional activity and also in the case of agriculture-related companies. CSR-oriented studies have a long history in the business and management literature; however, the agribusiness-related research papers and topics are underrepresented. Considering the diverse conflicts and disputes between the agribusiness and different social groups or stakeholders significant potential is associated with CSR-related issues. Due to global challenges, urbanization processes, climate-related issues and structural changes in agriculture the companies in agribusiness are more and more motivated to focus on CSR activities. The lack of in-depth analyses of climate-oriented CSR activities can be seen despite the large number of studies in relation to CSR issues. This analyse is focusing on a peculiar and pivotal topic namely the adaptation to climate change. The main results of the research can help managers in the agribusiness to be able to respond not only public pressure but global environmental and socio-economic challenges from the aspect of CSR. Moreover, to be able to tailor their CSR activities, it is better considering climate-related activities.

Based on the results it can be seen that the CSR consciousness of the companies is relatively undeveloped; however among the corporate values many mention the topic of sustainability. CSR communication is primarily an image factor. Companies are working to improve the image of them through the CSR communication. Companies in the agribusiness can take effective and efficient measures to reduce their carbon emissions by quantifying the carbon emissions associated and setting specific targets for the entire supply chain and each level of impact. Businesses must also have a clear strategy for achieving their goals, as well as indicators and measurement methods to monitor their compliance.

Climate change has a diverse and significant impact on the agricultural sector that is a pivotal research and policy area in Hungary. To reduce the possible impact of climate change the adaptation strategies are important and can play a significant role to be prepared for the forthcoming challenges not only an individual but company level as well. There are several actions which can help to reduce the climate change vulnerability in the agriculture such as irrigation, water harvesting, policy, using advanced technology, institutional framework, etc. There are mitigation measures that can be taken to avoid the increase in pollutant emissions such as energy efficiency, renewable energy, sustainable forest management, afforestation, reforestation, agroforestry, etc. The climate-oriented level of CSR activities was evaluated in the Hungarian agribusiness due to content analyses that showed a diverse picture and moderate level of interest concerning adaptation options and potential. There is a low level of financial and technology-oriented adaptation tools and activities among the examined top Hungarian companies. However, climate-oriented CSR activities in agribusiness can play a pivotal role in fostering the practical implementation and the steps towards sustainable agriculture in Hungary.

## Footnotes

1 Hungarian journal https://figyelo.hu/.

2 Energy sector: Energy industries, Manufacturing Industries and Construction, Transport, Other sectors, Fugitive Emissions from Fuels;

3 Industrial Processes and Product Use: Mineral Products, Chemical Industry, Metal Production, Non-energy Products from Fuels and Solvent Use, Electronics industry, Product Uses as Substitutes for ODS, Other Product Manufacture and Use;

4 The energy sector includes: Energy industries, Manufacturing Industries and Construction, Transport, Other sectors, Fugitive Emissions from Fuels.
